# Interferon-gamma drives programmed death-ligand 1 expression on islet β cells to limit T cell function during autoimmune diabetes

**DOI:** 10.1038/s41598-018-26471-9

**Published:** 2018-05-29

**Authors:** Kevin C. Osum, Adam L. Burrack, Tijana Martinov, Nathanael L. Sahli, Jason S. Mitchell, Christopher G. Tucker, Kristen E. Pauken, Klearchos Papas, Balamurugan Appakalai, Justin A. Spanier, Brian T. Fife

**Affiliations:** 1 Department of Medicine, Center for Immunology, University of Minnesota Medical School, Minneapolis, MN 55455 USA; 2 Department of Surgery, University of Arizona, Tucson, AZ USA; 3 Department of Surgery, University of Louisville, Louisville, KY USA

## Abstract

Type 1 diabetes is caused by autoreactive T cell-mediated β cell destruction. Even though co-inhibitory receptor programmed death-1 (PD-1) restrains autoimmunity, the expression and regulation of its cognate ligands on β cell remains unknown. Here, we interrogated β cell-intrinsic programmed death ligand-1 (PD-L1) expression in mouse and human islets. We measured a significant increase in the level of PD-L1 surface expression and the frequency of PD-L1^+^ β cells as non-obese diabetic (NOD) mice aged and developed diabetes. Increased β cell PD-L1 expression was dependent on T cell infiltration, as β cells from Rag1-deficient mice lacked PD-L1. Using Rag1-deficient NOD mouse islets, we determined that IFN-γ promotes β cell PD-L1 expression. We performed analogous experiments using human samples, and found a significant increase in β cell PD-L1 expression in type 1 diabetic samples compared to type 2 diabetic, autoantibody positive, and non-diabetic samples. Among type 1 diabetic samples, β cell PD-L1 expression correlated with insulitis. *In vitro* experiments with human islets from non-diabetic individuals showed that IFN-γ promoted β cell PD-L1 expression. These results suggest that insulin-producing β cells respond to pancreatic inflammation and IFN-γ production by upregulating PD-L1 expression to limit self-reactive T cells.

## Introduction

The inhibitory receptor Programmed Death-1 (PD-1) and its ligands Programmed Death Ligand (PD-L) 1 and 2 are critical regulators of immune cell function and autoimmunity^1–7^. Genetic deficiency of *PDCD1* in C57BL/6 and BALB/c mice leads to spontaneous lupus-like disease or autoimmune cardiomyopathy, respectively^5,7^, while non-obese diabetic (NOD) mice lacking either PD-1 or PD-L1 developed accelerated type 1 diabetes (T1D)^4,6^. Antibody blockade experiments suggest that PD-1:PD-L1 interactions, but not PD-1:PD-L2, are necessary for the maintenance of tolerance in the NOD model of T1D^8–14^. Several lines of evidence also suggest that the PD-1:PD-L1 pathway plays a role in maintaining islet tolerance in humans as recent onset patients with T1D have elevated gene expression levels of *CD274* (PD-L1) in whole-blood RNA analysis^15^. Additionally, single nucleotide polymorphisms in the *PDCD1* or *CD274* genes have been associated with T1D^16–18^. Finally, adverse events such as rapid autoimmunity including T1D can develop following checkpoint blockade in cancer patients^19,20^, further suggesting a role for this inhibitory pathway in autoimmunity.

PD-1 is rapidly expressed on the surface of T cells following activation, to diminish their proliferation and effector function upon ligand binding^21^. Many cells throughout the body can express PD-L1 including both hematopoietic and non-hematopoietic cells^22^. PD-L1 is constitutively expressed on resting T cells, B cells, dendritic cells, and macrophages, and is further upregulated upon cellular activation or in response to cytokines^1,23–25^. Previous work suggests that PD-1:PD-L1 interactions within the pancreas may limit autoimmune diabetes^6,8,26^. Despite this body of knowledge, the timing, location, and specific cellular interactions that are regulated by PD-1:PD-L1 in T1D remain unclear. While previous reports have shown intra-islet PD-L1 expression on infiltrating mononuclear cells^6,27^, and suggest a role for non-hematopoietic PD-L1 expression to limit diabetes, it is unclear if β cells themselves express PD-L1 and how this expression is regulated during diabetes progression. Additionally, enforcing PD-L1 expression on β cells under the insulin promoter has shown conflicting results, as NOD mice were protected from disease^28^ while diabetes-resistant mice were rendered susceptible with insulin promoter-driven PD-L1 expression^29^.

In this study, we measured islet β cell PD-L1 expression and regulation during diabetes pathogenesis. The goals of this study were to improve upon previous strategies for flow cytometric analysis of individual, insulin-positive, live β cells, and determine the specific regulators, location, and timing of PD-L1 expression in both mouse and human β cells. We utilized multicolor flow cytometry and epifluorescent microscopy to measure PD-L1 expression on islet β cells during spontaneous diabetes in NOD mice, and found that PD-L1 expression increased as mice approach diabetes onset, and was associated with islet infiltration. We also investigated the effect of cytokines on PD-L1 expression. The *CD274* promoter contains two interferon regulatory factor-1 (IRF-1) binding sites, and previous work has shown that type 1 and type 2 interferons (IFN) induce PD-L1 expression on T cells, B cells, endothelial cells, epithelial cells, and tumor cells^1,22^. We found that IFN-γ and to a lesser extent, IFN-α, promoted increased frequency of PD-L1^+^ β cells, and increased expression on a per cell basis. Similar to our findings in mice, within human pancreas we found that increased PD-L1 expression correlated with increased inflammatory T cell infiltration in pancreatic lesions. Interestingly, we observed a minor increase in PD-L1 staining in autoantibody positive patients in the absence of overt autoimmune diabetes and found that Th1-associated cytokine IFN-γ modulated PD-L1 expression *in vitro* on isolated human islets. Taken together, this work illustrates that both mouse and human islet β cells express PD-L1 in response to the same inflammatory cues, which may help delay islet destruction, but is ultimately insufficient to prevent β cell death.

## Results

### PD-L1 expression on islet β cells

**Figure 1:**
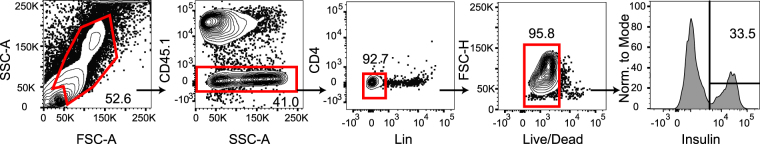
Intracellular insulin staining reliably detects β cells for further phenotypic analysis by flow cytometry. Shown is the gating strategy for pancreatic islets harvested from a 5 week NOD mouse. β cells were identified as forward and side scatter high cells, CD45.1^−^ cells, CD4^−^ and lineage negative (CD8^−^, CD11c^−^, CD11b^−^, B220^−^, F4/80^−^), live, intracellular insulin-positive cells.

**Figure 2:**
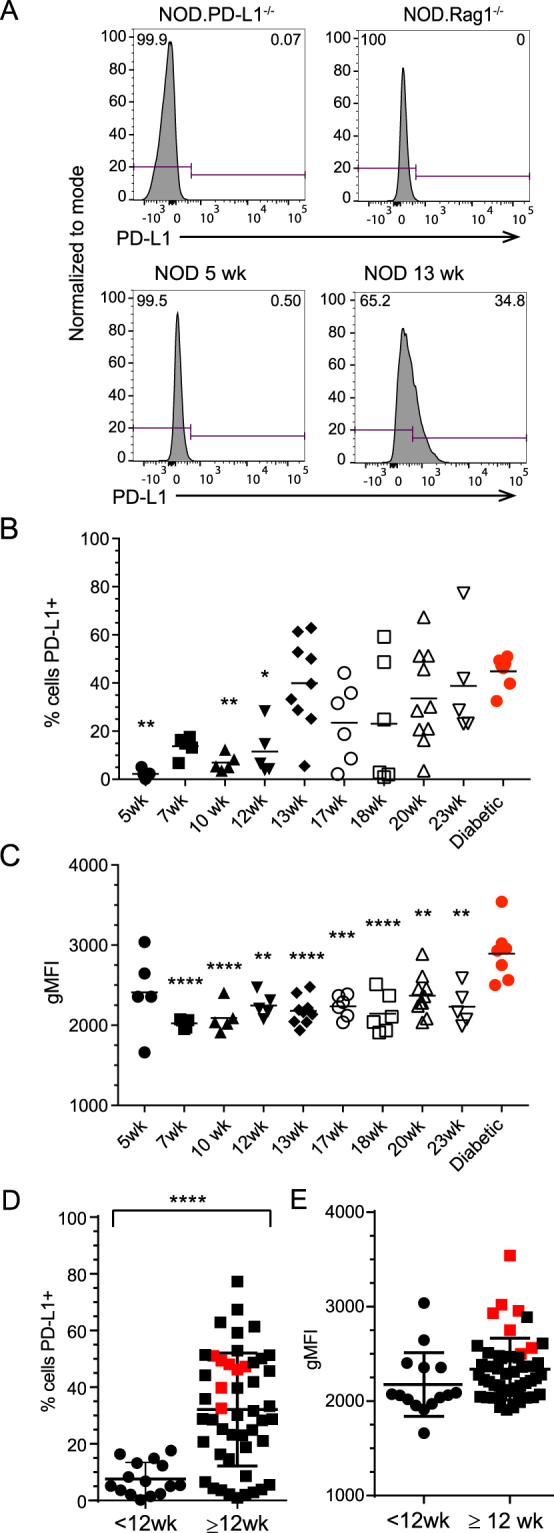
PD-L1 expression increases on β cells as NOD mice age and insulitis develops. To determine β cell PD-L1 expression in NOD mice, the gating scheme outlined in Fig. 1 was applied to dissociated NOD islets. (**A**) Shown are representative flow cytometry plots for control NOD.PD-L1^−/−^ mice, lymphocyte-deficient NOD.Rag1^−/−^ mice, and 5 or 13 week old NOD mice. NOD.PD-L1^−/−^ mouse islets were used to set negative and positive gates for PD-L1 staining. (**B**) Shown is the frequency PD-L1^+^ insulin-positive β cells from each mouse as a percentage of all live insulin positive β cells in 5–23 week old mice as indicated or from diabetic NOD mice. Each symbol represents an individual mouse (n = 68). The mean is shown with a black line. (**C**) The PD-L1 geometric mean fluorescence intensity (gMFI) from each mouse in (**B**), (n = 68). The mean is shown with a black line. (**D**) The percentage of PD-L1^+^ insulin-positive β cells from younger (<12 wks) and older (≥12 wks) NOD mice from (**B**). (**E**) PD-L1 gMFI of PD-L1^+^ insulin-positive β cells from younger (<12 wks) and older (≥12 wks) NOD mice from (**C**). The lines in (**D**,**E**) represent the mean ± standard deviation. Significance was determined as compared to diabetic mice: *p < 0.05, **p < 0.01, ***p < 0.001 using One-Way ANOVA with Tukey correction. Differences between the two age groups were analyzed using Student’s t-test (****p < 0.0001). Mice that were diabetic at the time of islet harvest are depicted in red (panels B–E).

We first performed a time course analysis of Programmed Death Ligand 1 (PD-L1) expression on islet β cells from the pancreas of NOD mice during type 1 diabetes. In NOD mice, β cells were identified as side and forward scatter high, CD45.1 negative, CD4 negative, lineage marker negative (CD8^−^, CD11c^−^, CD11b^−^, B220^−^, F4/80^−^), live cells, that were positive for intracellular insulin (Fig. 1). Using this strategy, we quantified PD-L1 expression on live β cells directly *ex vivo* from 5–23 week old non-diabetic NOD female mice and compared them to diabetic NOD female mice. Shown are representative plots from non-infiltrated 5 week old or 13-week old infiltrated non-diabetic NOD mice, and NOD.Rag1^−/−^ mice (Fig. 2A). NOD.PD-L1^−/−^ mice were used as negative controls for PD-L1 staining. We measured an increase in the percentage of PD-L1^+^ β cells as mice aged, with the highest frequency of PD-L1 in diabetic mice (Fig. 2B). Female NOD mice develop spontaneous autoimmune diabetes beginning at 12 weeks of age in our colony, with 70% incidence by 30 weeks of age (data not shown). Using 12 weeks as an age of demarcation, we demonstrate a statistically significant increase in the percentage of β cells expressing PD-L1 in NOD mice ≥12-weeks old (32.1 ± 2.8%) compared to young NOD mice (7.6 ± 1.5%, p < 0.0001, Fig. 2C). We also measured an increase in the level of PD-L1 expression on β cells as determined by the geometric mean fluorescent intensity (gMFI) on β cells from diabetic mice compared to all other ages except for 5 week old NOD mice (Fig. 2D). Again, using 12 weeks as an age of demarcation, we determined there was not a significant difference between the level of PD-L1 on islet β cells in NOD mice < or ≥12-weeks of age (Fig. 2E). Taken together, these results demonstrate a temporal correlation of PD-L1 expression with diabetes progression with the highest percentage of β cells expressing the highest levels of PD-L1 at diabetes onset.

### Islet β cell PD-L1 expression is associated with insulitis

**Figure 3:**
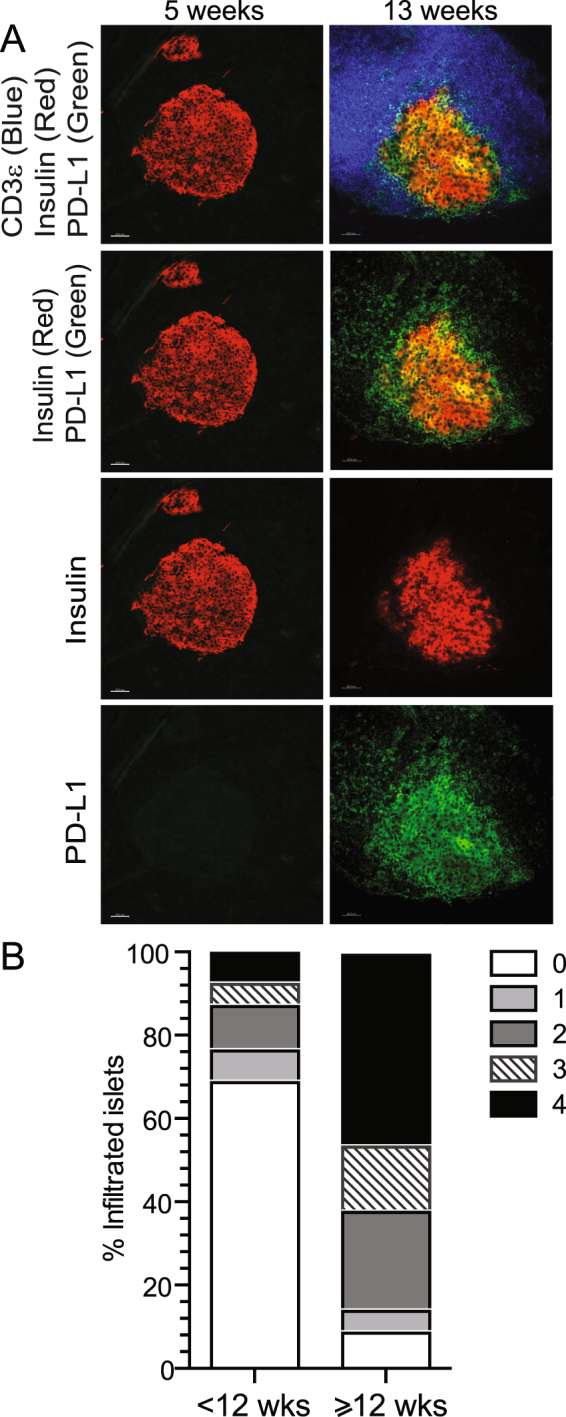
PD-L1 expression is concurrent with T cell infiltration within pancreatic islets in NOD mice. (**A**) PD-L1 expression and T cell infiltration in pancreatic islets from 5 and 13 week old NOD mice. Scale bar corresponds to 40 µm. (**B**) Cumulative insulitis scores from younger (<12 week) and older (≥12 week) NOD mice. Data represent >80 islets per age group, compiled from at least 4 independent experiments with 3–14 mice/experiment.

The absence of PD-L1 expression on islet β cells from NOD.Rag1^−/−^ mice and a significant increase in percent PD-L1 positive β cells after 12 weeks of age prompted us to determine if there was a correlation between PD-L1 expression and insulitis. It is well established that islet infiltration increases with age as mice approach diabetes onset^30,31^. We measured co-expression of PD-L1 and insulin in islets from 5 and 13-week old NOD mice by immunofluorescence (Fig. 3). The expression of islet PD-L1 was not detectable above background in 5-week old NOD mice, but widespread PD-L1 expression was present within islets from 13-week old NOD mice with notable overlap with insulin staining (represented as yellow, Fig. 3A). We detected few CD3ε^+^ T cells in the islets of 5 wk old NOD mice, whereas significant T cell pancreatic insulitis was seen in 13 wk old NOD mice (Fig. 3A). Again using 12 weeks of age as the cut off, we quantified the severity of insulitis using a five tier grading scale^30^. Results presented in Fig. 3B illustrate that approximately 25% of the islets in younger NOD mice (<12 wk) were infiltrated, with less than 10% having severe insulitis (score of 4). However, older NOD mice (≥12 wk) had significant and severe insulitis, with 88% of all islets classified as infiltrated and more than 50% of islets having severe insulitis (score of 4) (Fig. 3B). Despite the presence of insulitis in some mice younger than 12 weeks, PD-L1 was expressed at a low level and by few β cells (Fig. 3A).

### Islet β cell PD-L1 expression is driven by IFN-γ

**Figure 4:**
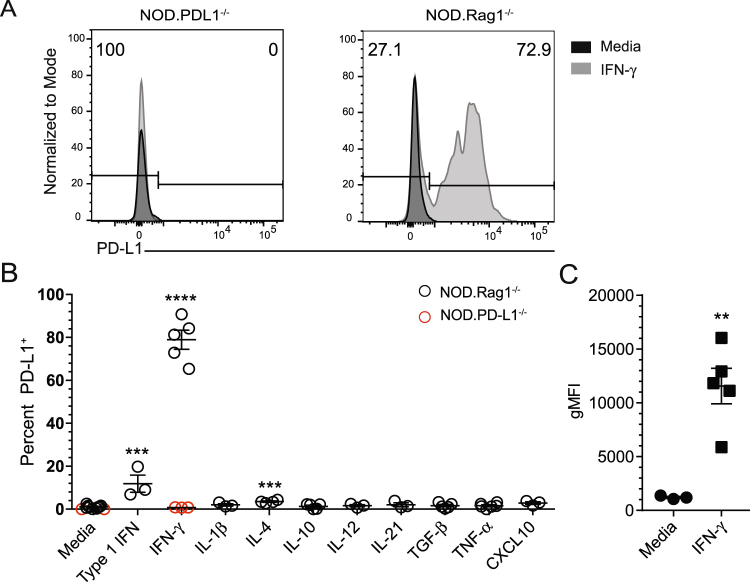
IFN-γ increases PD-L1 expression on mouse β cells. (**A**) Representative histogram plots of PD-L1 expression from pancreatic islets isolated from (left panel) NOD.Rag1^−/−^PD-L1^−/−^ negative control and (right panel) NOD.Rag1^−/−^ mice were cultured overnight with media alone or IFN-γ. (**B**) Frequency of PD-L1^+^ β cells isolated from NOD.Rag1^−/−^ (black) or NOD.PD-L1^−/−^ mice (red) after overnight culture with media alone or cytokines as indicated. (**C**) PD-L1 geometric mean fluorescence intensity (gMFI) of NOD.Rag1^−/−^ β cells cultured with media alone or IFN-γ from (**B**). Data are representative from 2–4 independent experiments and 3–5 mice/group for each experiment. The mean is shown with a black line ± standard deviation. Significance was determined by Student’s t-test. **p  <  0.01, ***p < 0.001, ****p < 0.0001 compared to media alone.

Given the correlation between PD-L1 expression and T cell infiltration, we next tested the role of cytokines in regulating PD-L1 expression on islet β cells. It has been reported that CD4^+^ T cells are required for the initiation of diabetes and that the Th1 cytokines IFN-γ and TNF-α may contribute to islet β cell death^32–36^. We hypothesized that exposing NOD islets to inflammatory cytokines or chemokines could provoke PD-L1 expression. We harvested NOD.Rag1^−/−^ islets and treated intact islets *in vitro* for 24 hours with IFN-α, IFN-γ, IL-1β, IL-4, IL-10, IL-12, IL-21, TGFβ, TNF-α, and CXCL10 and measured PD-L1 expression^1,30,31^. Following culture, islets were dissociated into single cells and PD-L1 expression on live beta cells was determined by flow cytometry according to the gating strategy shown in Fig. 1. Representative histogram plots from NOD.PD-L1^−/−^ mice are shown as negative controls (Fig. 4A, left). Rag1^−/−^ islets cultured in media alone do not express PD-L1 (Fig. 4A, right), while β cells cultured with IFN-γ show significant PD-L1 expression (Fig. 4A, right). Examination of the cytokine panel revealed that three cytokines induced PD-L1 expression. IFN-α and IL-4 caused a small, but significant increase in β cell PD-L1 expression with 11.9 ± 6.9% and 3.5 ± 0.65% β cells being PD-L1^+^ after culture, respectively (Fig. 4B). IFN-γ caused the most β cells to express PD-L1, with 78.92 ± 9.92% PD-L1^+^ β cells after culture (Fig. 4A and B, p < 0.0001). In addition, PD-L1 expression levels as measured by gMFI were significantly increased over media control after culture with IFN-γ (Fig. 4C, p = 0.0033). Taken together, these data demonstrate the Th1 cytokine IFN-γ caused β cells to express high levels of PD-L1.

### β cells from infiltrated diabetic patient pancreas sections express PD-L1

**Figure 5:**
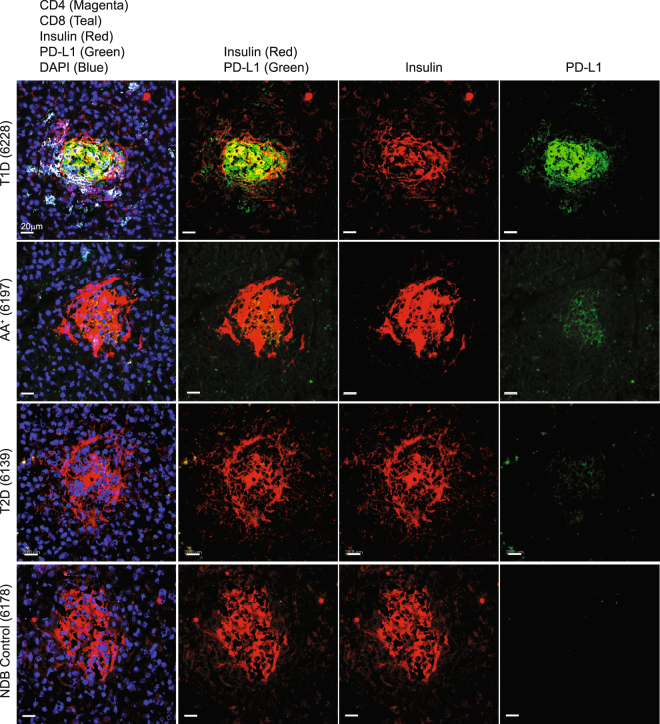
Human pancreas tissue from type 1 diabetic subjects express PD-L1. Human pancreas sections from type 1 diabetic (T1D), autoantibody positive (AA^+^), type 2 diabetic (T2D), and non-diabetic controls (NDB) were obtained from the Network for Pancreatic Organ Donation (nPOD) and stained for T cell markers (CD4, CD8), insulin, and PD-L1. Shown are representative islets from each group with 7–15 unique islets analyzed from three independent experiments with one patient per group. Scale bar corresponds to 20 µm.

**Figure 6:**
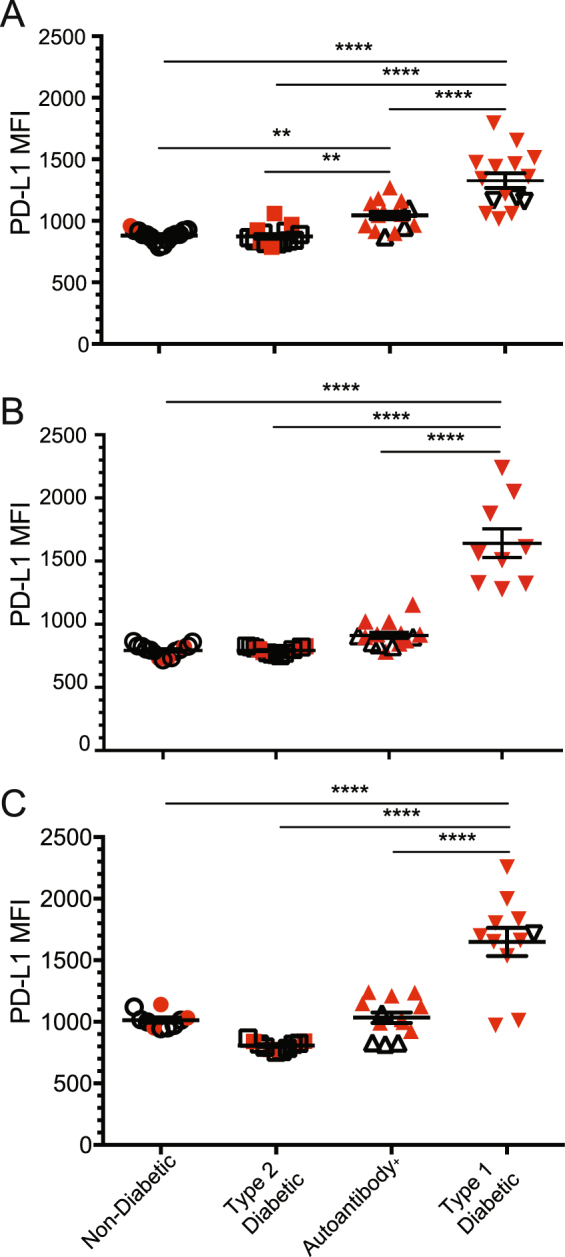
PD-L1 expression from pancreas β cell tissue is highest in T1D patients compared to autoantibody positive or T2D patients. β cell-intrinsic PD-L1 expression was quantified by epifluorescent microscopy for one subject in each group (non-diabetic, type 2 diabetic, autoantibody positive, type 1 diabetic) over three independent staining experiments (**A**–**C**). Each pancreatic islet from each subject is represented by one symbol, with red symbols representing islets containing T cell infiltration. Significant increase in islet PD-L1 mean fluorescence intensity (MFI) was determined by Student’s t-test within each experiment and is represented with: **p < 0.01, ***p < 0.001, ****p < 0.0001 as compared to non-T1D individuals.

The strong response to inflammation combined with Th1 cytokines in mouse islets lead us to hypothesize that diabetic human pancreatic sections would also exhibit significantly higher PD-L1 than non-diabetic control samples. We also predicted that sections from patients with type 2 diabetes would lack T cell infiltration and therefore lack PD-L1 expression given the proposed differences in mechanisms of β cell death in type 1 versus type 2 diabetes^37^. To test this, we obtained fresh frozen pancreas samples from the Network for Pancreatic Organ Donors with Diabetes (nPOD). These included sections from patients with type 1 diabetes, autoantibody positive individuals, patients with type 2 diabetes, and non-diabetic controls. Evaluation of these samples by epifluorescent microscopy allowed us to simultaneously evaluate insulitis, defined by the intra-islet presence of one or more CD4^+^ or CD8^+^ T cells, and determine the intensity of PD-L1 expression on islet β cells defined by insulin positive cells^38^ (Fig. 5). We quantified the expression of PD-L1 in three independent experiments with donors of multiple backgrounds and duration of diabetes (Supplemental Table 1). In all three experiments, islet beta cells from diabetic patients exhibited significantly higher PD-L1 expression on a per cell basis (Fig. 6A–C) compared to autoantibody positive individuals, patients with type 2 diabetes and non-diabetic individuals (representative images shown in Fig. 5). Given that only the islets from patients with T1D had significant insulitis, these results suggest that PD-L1 expression in human islets is associated with pancreas T cell infiltration, consistent with our analysis of NOD mouse islets.

### Human islet β cells upregulate PD-L1 in response to IFN-γ

**Figure 7:**
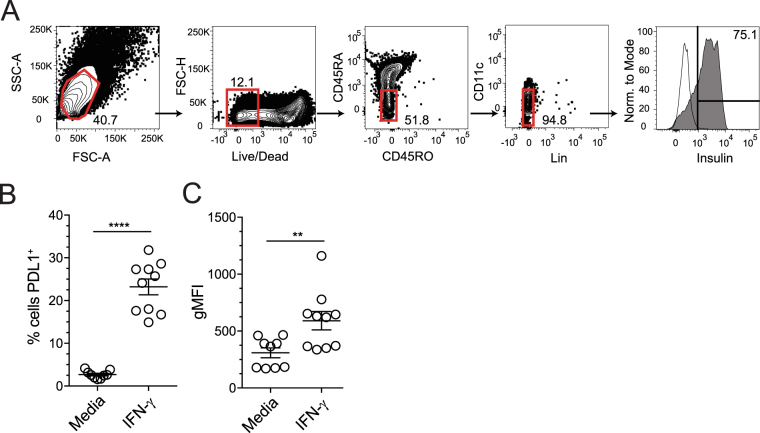
IFN-γ promotes PD-L1 expression on cultured human β cells. Cadaveric human islets from non-diabetic deceased donors were hand picked into 1000-islet aliquots and cultured overnight in the presence of media alone or media with IFN-γ. (**A**) Single cell flow cytometric analysis with antibodies for human CD45, CD11c, insulin, PD-L1 and live/dead stain was conducted in an analogous manner to experiments with murine islets in Fig. 4. Specifically, β cells were identified as forward and side scatter high cells, live cells, CD45RA/RO^−^, CD11c^−^ lineage negative (CD3^−^, CD11b^−^, CD11c^−^, CD20^−^), intracellular insulin-positive cells. Black solid line represents fluorescence minus one control for the insulin stain. (**B**) Frequency of PD-L1^+^ β cells with media alone, or media and IFN-γ and (**C**) PD-L1 geometric mean fluorescence intensity (gMFI). Data are pooled from 3 independent experiments, (n = 9–10). Significance was determined by Student’s t-test, **p < 0.01 and ****p < 0.0001.

Finally, we tested whether inflammatory cytokines could promote PD-L1 expression on human islet β cells. Given our cytokine results from mouse islets, and limited human islet availability, we restricted our analysis to the Th1 cytokine IFN-γ. Following 24 hours of culture, 1000 hand-counted human islets per well were dispersed to single cells and stained with a similar antibody panel as in the mouse studies to evaluate PD-L1 expression on β cells (Fig. 7A). Specifically, β cells were identified as forward and side scatter high cells, live cells, CD45RA/RO^−^, CD11c^−^ lineage negative (CD3^−^, CD11b^−^, CD11c^−^, CD20^−^), intracellular insulin-positive cells. Similar to our results using murine islets, Fig. 7B illustrates human β cells cultured in media alone did not express PD-L1 (4.3 ± 0.7%). We found that IFN-γ promoted a strong increase in the frequency of PD-L1^+^ β cells (23.2 ± 1.8%, p < 0.0001; Fig. 7B) along with enhanced PD-L1 expression on a per β cell basis (p = 0.0084; Fig. 7C). These data illustrate that NOD mouse and human islet β cells both respond to inflammatory cytokines and suggest that there may be common pro-inflammatory cytokine cue that regulates PD-L1 expression, in particular IFN-γ.

## Discussion

Previous work has demonstrated PD-L1 expression within the islets^6,27^, but the exact cellular source, timing, and regulation of PD-L1 remained unclear. In the current study, we designed a flow cytometric approach to detect live, insulin-producing β cells to evaluate PD-L1 expression during diabetes pathogenesis. Importantly, this flow cytometry-based approach allowed for exclusion of potentially contaminating intra-islet cell types and facilitated analysis of both murine and human β cell PD-L1 expression either directly *ex vivo* or following *in vitro* culture with cytokines. Using this approach, we determined that β cell PD-L1 expression increased as diabetes progressed. We also confirmed that increased PD-L1 expression was associated with lymphocyte infiltration in the context of spontaneous diabetes and following adoptive transfer of *in vitro* activated BDC2.5 CD4^+^ T cells (data not shown). We extended our analysis to human islets from non-diabetic, type 2 diabetic, autoantibody positive, and type 1 diabetic human patients and determined that β cells from type 1 diabetic and autoantibody positive patient pancreas sections also have significant PD-L1 associated with T cell infiltration.

The histological data (both mouse and human) suggests that islet β cell expression of PD-L1 is highly dependent on T cell infiltration. We demonstrate that inflamed islets have the highest level of PD-L1 expression and neighboring islets without significant T cell infiltration do not have strong PD-L1 expression. This result suggests that the cytokines driving PD-L1 act locally and not through a diffuse network, reminiscent of observations in human malignancies where PD-L1 expression is often detected on tumor cells adjacent to immune cell infiltrates^39^. This intimate regulation may also limit effective T cell inhibition globally throughout the pancreas and account for continued β cell death and eventual loss of islets. It is also true that PD-L1 expression is not uniquely expressed on islet β cells within the pancreas. Results in Fig. 5 illustrate PD-L1 staining that does not entirely co-localize with insulin. In other words, PD-L1 may be expressed on β cells that no longer make insulin, or may be expressed by intra-islet T cells, B cells, macrophages and dendritic cells. Previous work in the field has demonstrated islet PD-L1 expression was important in the transplant setting, and suggested that intra-islet dendritic cells or islet β cells were responsible for enhanced graft survival^27,40,41^. Our work suggests that β cell-intrinsic PD-L1 expression is strongly associated with insulitis and driven by IFN-γ and to a lesser extent IFN-α, and likely has a functional significance during disease progression. It is possible that other cytokines involved in T1D but not tested herein also drive PD-L1 expression. For instance, IL-17 has been shown to induce PD-L1 on human colon and prostate cancer cell lines in an NF-κB-dependent manner^42^. Whether IL-17 has the same effect on β cells remains to be tested.

Checkpoint blockade targeting the PD-1/PD-L1 pathway has garnered international attention as a promising cancer immunotherapy^43^. PD-1 is highly expressed on tumor infiltrating T cells and checkpoint therapy in patients with PD-L1^+^ tumors has been shown to be effective in early clinical trials^43^. However, adverse events such as rapid autoimmunity including T1D can develop following checkpoint blockade, further suggesting a role for this inhibitory pathway in regulating autoimmunity^19^. Therefore, given the current information and PD-L1 expression within the islets, and our previous work showing that nearly all islet antigen-specific CD4^+^ T cells express PD-1 in the pancreas^44^, cautious application of PD-1:PD-L1 blockade is warranted. Targeting tumor-specific T cells with checkpoint therapy while restraining autoimmunity is of great importance. Our work suggests that regulation of inflammatory cytokines may be a new avenue of focus to enhance or diminish PD-L1 mediated T cell inhibition, especially when considering checkpoint therapy in individuals with HLA types associated with higher risk of developing autoimmunity or individuals that test positive for islet specific autoantibodies. In one of the three human patients with increased autoantibodies we found a significant increase in PD-L1 expression over control and T2D patients (Fig. 6). PD-L1 expression status therefore could be used as an additional criterion prior to checkpoint inhibition and biomarker of adverse events following therapy.

PD-L1 expression by nonhematopoietic cells has been shown to limit immunopathology in the context of chronic viral infection^45–48^. The present study indicates that both mouse and human β cells may be able to reduce local effector T cell function and act in their own defense by expressing PD-L1 in response to IFN-γ. It is tempting to speculate that β cell-intrinsic PD-L1 expression plays a role in promoting T cell exhaustion in the pancreas. Our findings represent a significant advancement in our understanding of PD-L1 expression and regulation in response to inflammation in both animal models of diabetes and in autoantibody positive individuals or patients with T1D. However, even though PD-L1 can hold autoreactive T cells in check for some time, ultimately the majority of NOD female mice develop diabetes, indicating PD-L1 alone is not sufficient to prevent autoimmunity. Our findings in autoantibody positive and type 1 diabetic human samples support this hypothesis. Future studies using combinatorial approaches to limit autoreactive T cells together with enhancing β cell PD-L1 expression could provide a therapeutic opportunity to maintain β cell mass in patients with T1D.

## Methods

### Mice

NOD.Rag1^−/−^ and NOD.PD-L1^−/−^ mice were purchased from Jackson laboratories (Bar Harbor, ME). NOD mice were purchased from Taconic (Hudson, NY). Diabetes was defined as 2 consecutive blood glucose readings >250 mg/dL. All mice were housed in specific-pathogen free barrier facilities and all animal experiments and methods were performed in accordance with the relevant guidelines and regulations approved by the Institutional Animal Care and Use Committee at the University of Minnesota.

### Immunohistochemistry

Pancreata were harvested and prepared as described^49^. To identify PD-L1 expression on β cells, we used the following antibodies: goat anti-mouse PD-L1 (AF1019, Biotechne, Minneapolis, MN) at 1:500, AF647 bovine anti-goat (#805605180, Jackson ImmunoResearch, West Grove, PA) at 1:500, guinea pig anti-insulin (#A0564, Dako, Agilent, Santa Clara, CA) at 1:800, anti-guinea pig IgG (H + L) Cy3 (#706166148, Jackson ImmunoResearch) at 1:500, and anti-CD4 BV421 GK1.5 (BD Biosciences, Franklin Lakes, NJ) at 1:100. To determine the extent of insulitis, we stained sections with guinea pig anti-insulin (Dako) at 1:1000, anti-guinea pig IgG (H + L) Cy3 or Cy5 (#706175148, Jackson ImmunoResearch) at 1:1000, anti-CD4 AF488 GK1.5 at 1:100 and anti-CD8 APC or PE 53–6.7 (Thermo Fisher Scientific, Waltham, MA) at 1:100. Slides were mounted with Prolong Gold anti-fade with or without DAPI (Thermo Fisher Scientific). Images were acquired on a Leica DM6000B epifluorescent microscope. Islets were scored by a blinded investigator using the following scale: 0 – no insulitis, 1 – peri-insulitis, 2 – less than 25% of islet mass infiltrated, 3 – less than 75% of islet mass infiltrated, 4 – more than 75% of islet mass infiltrated^30^.

OCT-embedded fresh frozen human pancreas samples were provided from the Network for Pancreatic Organ Donors with Diabetes (nPOD). De-identified patient information and demographics is listed in Supplemental Table 1. Sections were prepared as above and stained with guinea pig anti-insulin (#A0564, Dako) at 1:800, anti-CD4 AF488 OKT4 (Biolegend, San Diego, CA) at 1:100, anti-CD8 BV480 SK1 (BD Biosciences) at 1:5000, anti-guinea pig IgG (H + L) Cy3 at 1:500 and anti-PD-L1 AF647 (D8T4X, Cell Signaling Technologies, Danvers, MA) at 1:100. Sections were mounted with Prolong Gold with DAPI. Images were acquired on Leica DM6000B epifluorescent microscope and analyzed using Imaris software (Version 8.4.1, Bitplane, South Windsor, CT). Surfaces were generated using the insulin channel and the mean fluorescent intensity (MFI) for PD-L1 was calculated using Imaris.

### Mouse pancreatic islet harvest and cytokine culture

Islets were harvested from NOD.Rag1^−/−^ female mice between 7–13 weeks of age, or 5–23 week old NOD mice, as previously described^50^. Briefly, 3 ml of ice-cold CIzyme (#005-1030, VitaCyte, Indianapolis, IN) was prepared following manufacturer’s instructions and injected into the common bile duct to inflate the donor pancreata. Pancreata were digested at 37 °C for 17 minutes. Following digestion, islets were washed with HBSS, centrifuged at room temperature at 2000 rpm for 20 min in Lympholyte1.1 (Cedarlane, Burlington, Ontario, CA), washed with 10% fetal calf serum (FCS) (Omega Scientific Inc, Tarzana, CA) in HBSS, and hand-counted. Islets were cultured in CMRL 1066 (Thermo Fisher Scientific) containing 1% Glutamax (Thermo Fisher Scientific), 10% FCS and 1% Pen/Strep (Thermo Fisher Scientific). To determine β cell response to pro-inflammatory cytokines, we supplemented cultures of 100 hand-counted pancreatic islets with murine IFN-γ [50 ng/ml], TNF-α [10 ng/ml], IL-1β [17.5 ng/ml], CXCL10 [20 ng/ml], IL-10 [20 ng/ml], TGF-β [10 ng/ml], IFN-α [10 ng/ml], IL-4 [10 ng/ml], IL-12 [20 ng/ml], and IL-21 [20 ng/ml] in non-tissue culture treated 12-well plates as indicated. All cytokines were purchased from Biotechne (Minneapolis, MN).

### Human islet preparations and cytokine culture

In compliance with federal regulations, islet isolations were performed at the University of Arizona or the University of Louisville utilizing pancreata from deceased donors with research consent. The islet isolation and culture procedures were performed as previously described^51,52^. Human islets were hand-counted and 1000 islets/well were cultured *in vitro* in CMRL 1066 containing 1% Glutamax, 1% Pen/Strep, 10% pooled human serum with or without human IFN-γ [50 ng/ml] (Biotechne) as indicated. Following overnight incubation at 37 °C containing 5% CO_2_, islets were dissociated using an 18-gauge needle on 1 ml syringes (BD Biosciences) in non-enzymatic cell dissociation solution (C5914, Sigma-Aldrich, St. Louis, MO). Cells were incubated with Human BD Fc block (# 564219 BD Biosciences) diluted in FACS buffer (2% FCS in PBS) prior to staining.

### Flow Cytometry

Single-cell suspensions of mouse pancreatic islets were blocked with 2.4G2 in FACS buffer and stained with: anti-CD4 BV650 (GK1.5, BD Biosciences), anti-CD45.1 APC (A20, BD Biosciences), anti-CD11b APC-efluor780 (M1/70), anti-B220 APC-efluor780 (RA3-6B2), anti-F4/80 APC-efluor780 (BM8), anti-CD8a APC-efluor780 (53–6.7), anti-CD11c PE-Cy7 (N418), anti-PD-L1 PerCP-efluor710 (MIH5) purchased from Thermo Fisher Scientific, and live/dead BV510 (Tonbo Biosciences, San Diego, CA)^53^. Following fixation and permeabilization (Tonbo Biosciences), cells were stained with guinea pig anti-insulin (#A0564, Dako) at 1:500, then washed and stained with secondary anti-guinea pig IgG (H + L) Cy3 at 1:200.

Single-cell suspensions of human pancreatic islets were stained with 5 µl per test of the following antibodies: anti-CD45.RA BV510 (HI100), anti-CD3 PE-Cy7 (SK7) purchased from Biolegend, live/dead ACP-efluor780, anti-CD11b PE-Cy7 (ICRF44), anti-CD20 PE-Cy7 (2H7) purchased from Tonbo Biosciences, anti-CD45RO BV650 (UCHL1), anti-CD11c BUV395 (B-Ly6) from BD Biosciences, anti-PDL1 PerCP-efluor710 (MIH1) purchased from Thermo Fisher Scientific. Following fixation and permeabilization (Tonbo Biosciences), cells were stained with anti-insulin (#A0564, Dako) at 1:500, then washed and stained with secondary anti-guinea pig AF647 at 1:200 (Jackson ImmunoResearch). Data were acquired on FACS Fortessa X-20 flow cytometer (BD Biosciences) and analyzed with FlowJo version 10 (Flow Jo, LLC, Ashland, OR).

### Statistical Analysis

Data were analyzed using Prism 7 (GraphPad Software, La Jolla, CA). P-values shown for individual figures represent results of two-tailed Student’s T-test or One-Way ANOVA with Tukey post hoc correction between cytokine treatment groups, or between different age groups for percent PD-L1-expressing β cells and PD-L1 geometric mean fluorescent intensity.

### Data availability statement

The datasets generated during and/or analyzed during the current study are available from the corresponding author on reasonable request.

## Electronic supplementary material

Supplemental Table

## References

[1] Francisco LM, Sage PT, Sharpe AH (2010). The PD-1 pathway in tolerance and autoimmunity. Immunol Rev.

[2] Fife BT, Bluestone JA (2008). Control of peripheral T-cell tolerance and autoimmunity via the CTLA-4 and PD-1 pathways. Immunol Rev.

[3] Okazaki T, Honjo T (2007). PD-1 and PD-1 ligands: from discovery to clinical application. Int Immunol.

[4] Wang J (2005). Establishment of NOD-Pdcd1^−/−^ mice as an efficient animal model of type I diabetes. Proc Natl Acad Sci USA.

[5] Nishimura H, Nose M, Hiai H, Minato N, Honjo T (1999). Development of lupus-like autoimmune diseases by disruption of the PD-1 gene encoding an ITIM motif-carrying immunoreceptor. Immunity.

[6] Keir ME (2006). Tissue expression of PD-L1 mediates peripheral T cell tolerance. J Exp Med.

[7] Nishimura H (2001). Autoimmune dilated cardiomyopathy in PD-1 receptor-deficient mice. Science.

[8] Guleria I (2007). Mechanisms of PDL1-mediated regulation of autoimmune diabetes. Clin Immunol.

[9] Ansari MJ (2003). The programmed death-1 (PD-1) pathway regulates autoimmune diabetes in nonobese diabetic (NOD) mice. J Exp Med.

[10] Paterson AM (2011). The programmed death-1 ligand 1:B7-1 pathway restrains diabetogenic effector T cells *in vivo*. J Immunol.

[11] Fife BT (2006). Insulin-induced remission in new-onset NOD mice is maintained by the PD-1-PD-L1 pathway. J Exp Med.

[12] Fife BT (2009). Interactions between PD-1 and PD-L1 promote tolerance by blocking the TCR-induced stop signal. Nat Immunol.

[13] Jiang TT (2016). Programmed Death-1 Culls Peripheral Accumulation of High-Affinity Autoreactive CD4 T Cells to Protect against Autoimmunity. Cell Rep.

[14] Martinov, T., Spanier, J. A., Pauken, K. E. & Fife, B. T. PD-1 pathway-mediated regulation of islet-specific CD4+ T cell subsets in autoimmune diabetes. *Immunoendocrinology (Houst)* **3**, 10.14800/ie.1164 (2016). 10.14800/ie.1164 PMC5027981 27656680

[15] Fang, C. *et al*. Genome-wide gene expression profiling reveals that CD274 is up-regulated new-onset type 1 diabetes mellitus. *Acta Diabetol*, 10.1007/s00592-017-1005-y (2017). 10.1007/s00592-017-1005-y 28577136

[16] Nielsen C, Hansen D, Husby S, Jacobsen BB, Lillevang ST (2003). Association of a putative regulatory polymorphism in the PD-1 gene with susceptibility to type 1 diabetes. Tissue Antigens.

[17] Ni R (2007). PD-1 gene haplotype is associated with the development of type 1 diabetes mellitus in Japanese children. Hum Genet.

[18] Pizarro C (2014). PD-L1 gene polymorphisms and low serum level of PD-L1 protein are associated to type 1 diabetes in Chile. Diabetes/metabolism research and reviews.

[19] Byun DJ, Wolchok JD, Rosenberg LM, Girotra M (2017). Cancer immunotherapy - immune checkpoint blockade and associated endocrinopathies. Nature reviews. Endocrinology.

[20] Hughes J (2015). Precipitation of autoimmune diabetes with anti-PD-1 immunotherapy. Diabetes Care.

[21] Zamani MR, Aslani S, Salmaninejad A, Javan MR, Rezaei N (2016). PD-1/PD-L and autoimmunity: A growing relationship. Cell Immunol.

[22] Fife BT, Pauken KE (2011). The role of the PD-1 pathway in autoimmunity and peripheral tolerance. Ann N Y Acad Sci.

[23] Liang SC (2003). Regulation of PD-1, PD-L1, and PD-L2 expression during normal and autoimmune responses. Eur J Immunol.

[24] Freeman GJ (2000). Engagement of the PD-1 immunoinhibitory receptor by a novel B7 family member leads to negative regulation of lymphocyte activation. J Exp Med.

[25] Tamura H (2001). B7-H1 costimulation preferentially enhances CD28-independent T-helper cell function. Blood.

[26] Rui J (2017). Beta Cells that Resist Immunological Attack Develop during Progression of Autoimmune Diabetes in NOD Mice. Cell Metab.

[27] Keir ME, Freeman GJ, Sharpe AH (2007). PD-1 regulates self-reactive CD8+ T cell responses to antigen in lymph nodes and tissues. J Immunol.

[28] Wang CJ (2008). Protective role of programmed death 1 ligand 1 (PD-L1)in nonobese diabetic mice: the paradox in transgenic models. Diabetes.

[29] Subudhi SK (2004). Local expression of B7-H1 promotes organ-specific autoimmunity and transplant rejection. J Clin Invest.

[30] Leiter, E. H. The NOD mouse: a model for insulin-dependent diabetes mellitus. *Curr Protoc Immuno*l Chapter 15, Unit15 19, 10.1002/0471142735.im1509s24 (2001). 10.1002/0471142735.im1509s24 18432739

[31] Anderson MS, Bluestone JA (2005). The NOD mouse: a model of immune dysregulation. Annu Rev Immunol.

[32] Timsit J, Debray-Sachs M, Boitard C, Bach JF (1988). Cell-mediated immunity to pancreatic islet cells in the non-obese diabetic (NOD) mouse: *in vitro* characterization and time course study. Clin Exp Immunol.

[33] Wang Y, Hao L, Gill RG, Lafferty KJ (1987). Autoimmune diabetes in NOD mouse is L3T4 T-lymphocyte dependent. Diabetes.

[34] Shizuru JA, Taylor-Edwards C, Banks BA, Gregory AK, Fathman CG (1988). Immunotherapy of the nonobese diabetic mouse: treatment with an antibody to T-helper lymphocytes. Science.

[35] Suk K (2001). IFN-gamma/TNF-alpha synergism as the final effector in autoimmune diabetes: a key role for STAT1/IFN regulatory factor-1 pathway in pancreatic beta cell death. J Immunol.

[36] Cantor J, Haskins K (2007). Recruitment and activation of macrophages by pathogenic CD4 T cells in type 1 diabetes: evidence for involvement of CCR8 and CCL1. J Immunol.

[37] Cnop M (2005). Mechanisms of pancreatic beta-cell death in type 1 and type 2 diabetes: many differences, few similarities. Diabetes.

[38] Campbell-Thompson M (2016). Insulitis and beta-Cell Mass in the Natural History of Type 1 Diabetes. Diabetes.

[39] Topalian SL, Taube JM, Anders RA, Pardoll DM (2016). Mechanism-driven biomarkers to guide immune checkpoint blockade in cancer therapy. Nat Rev Cancer.

[40] Li T (2015). PD-1/PD-L1 costimulatory pathway-induced mouse islet transplantation immune tolerance. Transplant Proc.

[41] Baas M (2016). TGFbeta-dependent expression of PD-1 and PD-L1 controls CD8(+) T cell anergy in transplant tolerance. Elife.

[42] Wang X (2017). Inflammatory cytokines IL-17 and TNF-alpha up-regulate PD-L1 expression in human prostate and colon cancer cells. Immunol Lett.

[43] Postow MA, Callahan MK, Wolchok JD (2015). Immune Checkpoint Blockade in Cancer Therapy. J Clin Oncol.

[44] Pauken KE (2015). Cutting edge: identification of autoreactive CD4+ and CD8+ T cell subsets resistant to PD-1 pathway blockade. J Immunol.

[45] Mueller SN (2010). PD-L1 has distinct functions in hematopoietic and nonhematopoietic cells in regulating T cell responses during chronic infection in mice. J Clin Invest.

[46] Seko Y, Yagita H, Okumura K, Azuma M, Nagai R (2007). Roles of programmed death-1 (PD-1)/PD-1 ligands pathway in the development of murine acute myocarditis caused by coxsackievirus B3. Cardiovasc Res.

[47] Filippi CM, Estes EA, Oldham JE, von Herrath MG (2009). Immunoregulatory mechanisms triggered by viral infections protect from type 1 diabetes in mice. J Clin Invest.

[48] Akhmetzyanova I (2015). PD-L1 Expression on Retrovirus-Infected Cells Mediates Immune Escape from CD8+ T Cell Killing. PLoS pathogens.

[49] Pauken KE, Jenkins MK, Azuma M, Fife BT (2013). PD-1, but not PD-L1, expressed by islet-reactive CD4+ T cells suppresses infiltration of the pancreas during type 1 diabetes. Diabetes.

[50] Bishop NH, Nelsen MK, Beard KS, Coulombe M, Gill RG (2017). Differential Impact of Chronic Hyperglycemia on Humoral Versus Cellular Primary Alloimmunity. Diabetes.

[51] Ricordi C (2016). National Institutes of Health-Sponsored Clinical Islet Transplantation Consortium Phase 3 Trial: Manufacture of a Complex Cellular Product at Eight Processing Facilities. Diabetes.

[52] Balamurugan AN (2016). Identifying Effective Enzyme Activity Targets for Recombinant Class I and Class II Collagenase for Successful Human Islet Isolation. Transplant Direct.

[53] Pauken KE (2013). Cutting edge: type 1 diabetes occurs despite robust anergy among endogenous insulin-specific CD4 T cells in NOD mice. J Immunol.

