# Supplementary material for: Interferon-gamma drives programmed death-ligand 1 expression on islet β cells to limit T cell function during autoimmune diabetes
Source: Sci Rep. 2018 May 29;8:8295. doi: 10.1038/s41598-018-26471-9 (PMC5974126; doi:10.1038/s41598-018-26471-9)
Supplement: Supplementary file 1 — Supplemental Table [file 41598_2018_26471_MOESM1_ESM.pdf]

Supplemental Table Patient Demographics for panels in Figure 6.								
Panel A	Type	Age	Diabetes duration (years)	Gender	Ethnicity	BMI	HbA1c	C peptide
**6271**	**No diabetes**	**17**		**Male**	**Caucasian**	**24.4**		**11.47**
**6139**	**T2D**	**37.2**	**1.5**	**Female**	**Hispanic/Latino**	**45.4**		**0.6**
**6267**	**Auto Ab Pos**	**23**		**Female**	**Caucasian**	**23.5**	**5**	**16.59**
**6247**	**T1D**	**24**	**0.6**	**Male**	**Caucasian**	**24.27**		**0.47**
Panel B	Type	Age	Diabetes duration (years)	Gender	Ethnicity	BMI	HbA1c	C peptide
**6178 02**	**No diabetes**	**24.5**		**Female**	**Caucasian**	**27.5**	**5**	**4.55**
**6059 06**	**T2D**	**18.8**	**0.25**	**Female**	**Hispanic/Latino**	**39.3**		**10.68**
**6197 03**	**Auto Ab Pos**	**22**		**Male**	**African American**	**28.2**	**5.5**	**17.48**
**6228 01**	**T1D**	**13**	**0**	**Male**	**Caucasian**	**17.36**	**13.3**	**0.1**
Panel C	Type	Age	Diabetes duration (years)	Gender	Ethnicity	BMI	HbA1c	C peptide
**6386**	**No diabetes**	**14**		**Male**	**Caucasian**	**23.89**	**5.6**	**1.12**
**6308**	**T2D**	**13**	**1**	**Female**	**Caucasian**	**34.1**		**5.2**
**6310**	**Auto Ab Pos**	**28**		**Female**	**Hispanic/Latino**	**22.4**		**10.54**
**6362**	**T1D**	**24.9**	**0**	**Male**	**Caucasian**	**28.5**	**10**	**0.38**

Interferon-gamma drives programmed death-ligand 1 expression on islet β cells to limit T cell function during autoimmune diabetes

Kevin C. Osum[1, §], Adam L. Burrack[1, §], Tijana Martinov[1,§], Nathanael L. Sahli[1], Jason S. Mitchell[1], Christopher G. Tucker[1], Kristen E. Pauken[1], Klearchos Papas[2], Balamurugan Appakalia[3], Justin A. Spanier[1], and Brian T. Fife[1,*]

[§]Authors contributed equally.
